# Pt thin-film resistance thermo detectors with stable interfaces for potential integration in SiC high-temperature pressure sensors

**DOI:** 10.1038/s41378-024-00746-w

**Published:** 2024-09-26

**Authors:** Ziyan Fang, Xiaoyu Wu, Hu Zhao, Xudong Fang, Chen Wu, Dong Zhang, Zhongkai Zhang, Bian Tian, Libo Zhao, Tiefu Li, Prateek Verma, Ryutaro Maeda, Zhuangde Jiang

**Affiliations:** 1 State Key Laboratory for Manufacturing Systems Engineering, International Joint Laboratory for Micro/Nano Manufacturing and Measurement Technologies, Xi’an Jiaotong University, Xi’an, 710049 China; 2 School of Mechanical Engineering, Xi’an Jiaotong University, Xi’an, 710049 China; 3 Shandong Laboratory of Yantai Advanced Materials and Green Manufacturing at Yantai, Yantai, 264000 China; 4 Xi’an Jiaotong University (Yantai) Research Institute for Intelligent Sensing Technology and System, Xi’an, China; 5 School of Instrument Science and Technology, Xi’an Jiaotong University, Xi’an, 710049 China; 6 School of Integrated Circuits, Tsinghua University, Beijing, 100084 China; 7 Department of Chemical Engineering, University of Arkansas, Fayetteville, AR 72701 USA

**Keywords:** Electronic properties and materials, Electrical and electronic engineering

## Abstract

Due to the excellent mechanical, chemical, and electrical properties of third-generation semiconductor silicon carbide (SiC), pressure sensors utilizing this material might be able to operate in extreme environments with temperatures exceeding 300 °C. However, the significant output drift at elevated temperatures challenges the precision and stability of measurements. Real-time in situ temperature monitoring of the pressure sensor chip is highly important for the accurate compensation of the pressure sensor. In this study, we fabricate platinum (Pt) thin-film resistance temperature detectors (RTDs) on a SiC substrate by incorporating aluminum oxide (Al_2_O_3_) as the transition layer and utilizing aluminum nitride (AlN) grooves for alignment through microfabrication techniques. The composite layers strongly adhere to the substrate at temperatures reaching 950 °C, and the interface of the Al_2_O_3_/Pt bilayer remains stable at elevated temperatures of approximately 950 °C. This stability contributes to the excellent high-temperature electrical performance of the Pt RTD, enabling it to endure temperatures exceeding 850 °C with good linearity. These characteristics establish a basis for the future integration of Pt RTD in SiC pressure sensors. Furthermore, tests and analyses are conducted on the interfacial diffusion, surface morphological, microstructural, and electrical properties of the Pt films at various annealing temperatures. It can be inferred that the tensile stress and self-diffusion of Pt films lead to the formation of hillocks, ultimately reducing the electrical performance of the Pt thin-film RTD. To increase the upper temperature threshold, steps should be taken to prevent the agglomeration of Pt films.

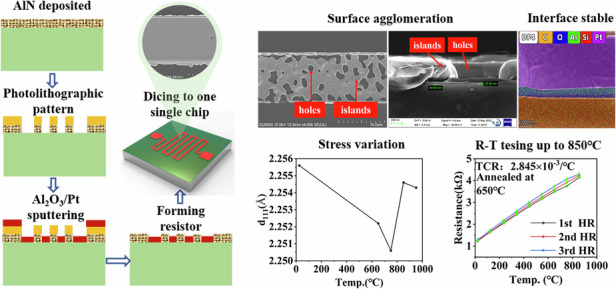

## Introduction

The aerospace, environmental, and automotive industries are often characterized by harsh environments featuring high temperatures and corrosion conditions. In aeroengines, the temperature and pressure of the combustion chamber can exceed 1000 °C and 1.5 MPa^1,2^, respectively. Real-time in situ monitoring of temperature and pressure parameters for critical components is highly important for material selection and failure analysis^3,4^. Traditional discrete devices have high costs and require large installation spaces. It is necessary to develop an on-chip integrated sensor for the simultaneous monitoring of temperature and pressure. Additionally, since conventional silicon-based pressure sensors cannot adapt to high temperatures exceeding 300 °C^5,6^, the development of high-temperature pressure sensors based on third-generation wide-bandgap semiconductor silicon carbide (SiC) has attracted considerable attention. However, the temperature drift characteristics of SiC-based pressure sensors severely affect the accuracy of the sensor^7^; thus, it is urgent to carry out the in situ temperature compensation of SiC-based pressure sensors. Based on the aforementioned applications, which involve the in situ monitoring of the temperatures of SiC pressure sensor chips and the compensation of the temperature drift characteristics of pressure sensors, it is necessary to conduct research on temperature sensors based on SiC wafers. This research lays the foundation for the on-chip integration of temperature sensors with SiC pressure sensors. In this paper, a temperature sensor based on SiC that is resistant to high temperatures is proposed for the first time.

There are several studies on the fabrication of temperature sensors using the temperature-sensitive properties of SiC, including thermistors based on SiC thin films and diode temperature detectors based on SiC. These devices can withstand temperatures that do not exceed 600 °C^8–11^. However, Pt resistors exhibit high precision and good linearity over a wide temperature range, are not easily oxidized and are easy to integrate with MEMS processes^12–15^. Therefore, Pt resistors are selected as the temperature sensing unit in this study. The aims of this paper are to elucidate the preparation processes of Pt RTD on SiC substrates and to explore its high-temperature performance.

When fabricating Pt thin-film resistors, it is common to add a metal adhesion layer, such as chromium, titanium, or tantalum^16,17^ to prevent the Pt thin film from detaching from the substrate. However, chromium, titanium, and tantalum adhesion layers oxidize or diffuse at 300, 450, and 600 °C, respectively^18–22^. In particular, the diffusion of the adhesion layer to the Pt film reduces the temperature coefficient of resistance (TCR) of the Pt RTD^23,24^ and promotes the deterioration of the surface of the Pt film^25,26^, which decreases its electrical performance and stability. Furthermore, the degradation of the adhesion layer promotes the shedding of the Pt film^23^, resulting in the direct removal of the Pt resistors. The interface diffusion between platinum (Pt) and the substrate degrades the electrical performance of Pt resistors^19,27,28^. Therefore, it is crucial to add a suitable intermediate layer between the Pt and the substrate to prevent diffusion between the Pt and the substrate and between the Pt and the intermediate layer to enhance the high-temperature resistance of the Pt.

Herein, a MEMS preparation method based on AlN groove-fixed Pt thin-film RTD using an Al_2_O_3_ intermediate layer was proposed. A Pt thin-film RTD was successfully prepared on a SiC substrate, and the interface between the Pt film and the Al_2_O_3_ intermediate layer was stable at high temperatures (reaching 950 °C), which promoted the good high-temperature electrical performance of the RTD. The surface morphologies of the Pt films on the SiC substrates at high temperatures, the interfacial diffusion between the Pt film and the substrate, and the electrical properties of the Pt thin-film RTD were examined. Correlations between the electrical properties, changes in the surface morphology, and microscopic stresses of the Pt thin films were established. The reasons for the poor stability and repeatability of Pt RTD were revealed microscopically.

## Experimental procedures

### Sample preparation

**Fig. 1:**
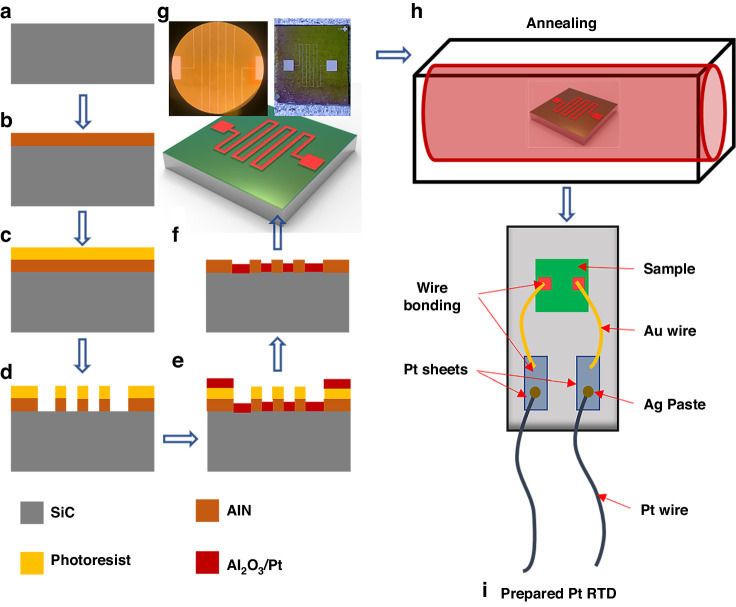
Process flow diagram for the fabrication of Pt thin films RTD. **a** SiC wafer cleaning using standard RCA procedures; **b** sputtering of an AlN film on a SiC substrate; **c** homogenization of photoresists; **d** lithographic patterning of sensitive resistance strips; **e** sputtering of Al_2_O_3_/Pt; **f** removal of the photoresist; **g** image of a single chip; **h** thermal annealing of the chip in a tube furnace; and **i** packaging of the chip for performance tests

In this work, Al_2_O_3_ was proposed as an intermediate layer between the Pt resistor and the SiC substrate. However, due to the weak adhesion between the Pt thin film and the Al_2_O_3_ film, which could detach the Pt thin-film resistor, a microfabrication process was developed. This process utilized grooves in the AlN film to securely anchor the Pt thin-film resistors, as illustrated in Fig. 1. Four-inch 4H-SiC substrates, each with a thickness of 350 µm, were purchased from TanKeBlue Semiconductor Co., Ltd. The substrate underwent a series of processes beginning with the deposition of an AlN film via magnetron sputtering (Fig. 1b). Homogenization was achieved using an EPG 535 photoresist (Fig. 1c). ZX-238 solution was used for development after ultraviolet exposure (Fig. 1d). After development, a photolithographic pattern of the Pt resistor shape was formed. Simultaneously, the AlN film was etched by the developer solution, creating grooves in the shape of a Pt resistor. These grooves were used to anchor the Pt thin-film resistors. The process continued with the deposition of the Al_2_O_3_ film and Pt film through radio frequency (RF) and direct current (DC) magnetron sputtering, respectively, without breaking the vacuum. The vacuum conditions were maintained at 4.3 ×10^−5^ Torr with an argon flow rate of 60 sccm, corresponding to a working pressure of 1.36 Pa (Fig. 1e). The divestiture step, which is shown in Fig. 1f, involved removing the photoresist to form Pt thin-film resistors using acetone as the peeling solution. The thickness of the Pt thin-film resistor was less than that of the AlN film. Therefore, the Pt thin-film resistor could be fully anchored within the grooves of the AlN without easily detaching. Thus, Pt thin-film RTDs were successfully prepared on SiC substrates. A schematic diagram of the structure of the Pt thin-film RTD, a physical diagram of a single-chip post-dicing device with dimensions of 7.5 mm × 7.5 mm, and an optical image showing distinct structural features are shown in Fig. 1g. The resistor width was 10 µm, and the linewidth error was less than ±1~2 µm. The chip underwent an annealing process in air with a heating rate of 10 °C/min, as detailed in Fig. 1h, for 1.5 h at various temperatures (650, 750, 850, and 950 °C). This process was followed by furnace cooling. Figure 1i shows the chip mounted on an alumina substrate for subsequent testing. The electrical connection between the chip and the external circuit involved three steps: initially, the chip and a Pt sheet were attached to the alumina substrate using high-temperature cement; then, gold wires were employed for electrical connection between the Pt pad on the chip and the Pt sheet; and finally, a high-temperature conductive silver paste was used to bond the Pt wire to the Pt sheet.

### Characterization

The interfacial diffusion and crystal orientation characteristics of the Pt films were characterized by Lorentz transmission electron microscopy (Talos F200X, Thermo Fisher Scientific, America). The sample was sliced by a focused ion beam (FIB) (Thermo Scientific Helios 5 UX, Thermo Scientific, The Czech Republic) and placed on a copper grid. The copper grid with the sample was inserted into a Lorentz transmission electron microscope and observed in high-angle annular dark field (HAADF) mode. The surface microtopographies of the Pt films were characterized by field-emission scanning electron microscopy (FE-SEM) (su-8010, Hita chi, Japan). The film surface morphology was observed in secondary electron mode with a voltage setting of 10 kV. The cross-sectional micromorphologies and thicknesses of the Al_2_O_3_/Pt double-layer films were characterized by FE-SEM (Gemini SEM 500, Zeiss, Germany). The cross-sectional morphology was observed, and the film thickness was measured in InLens mode with a voltage setting of 10 kV. The surface roughnesses of the Pt films were characterized by atomic force microscopy (AFM) (INNOVA, Bruker, America). The tap mode was used during AFM scanning. The scanning area was set to 20 µm × 20 µm. The crystal information of the Pt film was characterized by X-ray diffraction (XRD) (D8 ADVANCE, Bruker, USA). The X-ray source was a copper target with a wavelength of 0.15418 nm. Scanning was conducted in grazing incidence mode. The operating voltage was set to 40 V, with an operating current of 40 mA. The scanning angle ranged from 20° to 90° with a step size of 3417, and each step scan took 0.4 s. The square resistance of the Pt film was characterized by a four-point probe tester (HPS 2526, China). The measurements were conducted in point mode at medium speed. The sample size was 14 mm × 4.5 mm.

### Resistivity testing of Pt thin films

The Pt film samples examined for resistivity were from the same batch as those used for RTD, measuring 14 mm × 4.5 mm. The square resistance was characterized by a four-point probe tester, and the thickness was measured by FE-SEM. The resistivity was calculated by the following equation:

1 $${\rm{\rho }}={R}_{{sq}}\cdot h$$

where *R*_*sq*_ is the square resistance of the film in units of ohms per square and *h* is the thickness of the film in nm.

### Calculation of the grain size and crystal plane spacing of the Pt thin films

The grain size was estimated using the Scherrer formula^29^:

2 $$D=\frac{K\,\lambda }{\beta \; {\mathrm{cos}}\theta }$$

The crystal plane spacing was calculated using the Bragg diffraction formula:

3 $$d=\frac{\lambda }{2\;{\mathrm{sin}}\theta }$$

where *D* is the grain size; *λ* is the X-ray wavelength, and for ${K}_{\alpha }$ of Cu, *λ* is equal to 0.15406 nm; *K* is the Scherrer constant, which is equal to 0.89; *β* is the full width at half maximum; θ is the Bragg diffraction angle; and *d* is the crystal plane spacing.

### High-temperature electrical performance test of Pt thin-film RTD

A programmed high-temperature furnace (SXC-2-13G, Hangzhou Blue Sky Instrument Co., Ltd., China) was utilized to establish a high-temperature testing environment. Nine temperature points ranging from 0 to 800 °C, namely, 25 °C (ambient temperature), 100, 200, 300, 400, 500, 600, 700, and 800 °C, were set, and the heating rate was 5 °C/min. The samples were placed in the furnace, and temperature changes were monitored using an external K-type armored thermocouple. At each temperature point, the duration time was set to 30 min to ensure stable temperature conditions for the tests.

The resistance and temperature were recorded using a DAQ 6510 data collection and recording multimeter system (DAQ 6510, Tektronix, America). During the heating process mentioned above, nine temperature points and the corresponding resistance values were collected as the average of the temperature and resistance when the furnace temperature was stable (i.e., when the temperature error was less than 5%) during the 30-min holding period. The sensitivity and linearity (*R*^2^) of the Pt thin-film RTD were calculated. TCR, which is the sensitivity indicator of Pt thin-film RTD, was expressed as follows:

4 $${\rm{TCR}}=\frac{R-{R}_{0}}{{R}_{0}(T-{T}_{0})}$$

where *R*_0_ is the resistance at 0 °C. A larger TCR indicated a higher sensitivity of the Pt thin-film RTD.

## Results and discussion

SiC-based Pt thin-film RTDs were annealed at different temperatures to explore the effects of annealing temperature on the interfacial diffusion, stress, surface morphological, and electrical properties and the relationships among micromorphological, microscopic stress, and electrical properties.

### Microstructural analysis of Pt thin-film RTDs

#### Interfacial diffusion analysis

**Fig. 2:**
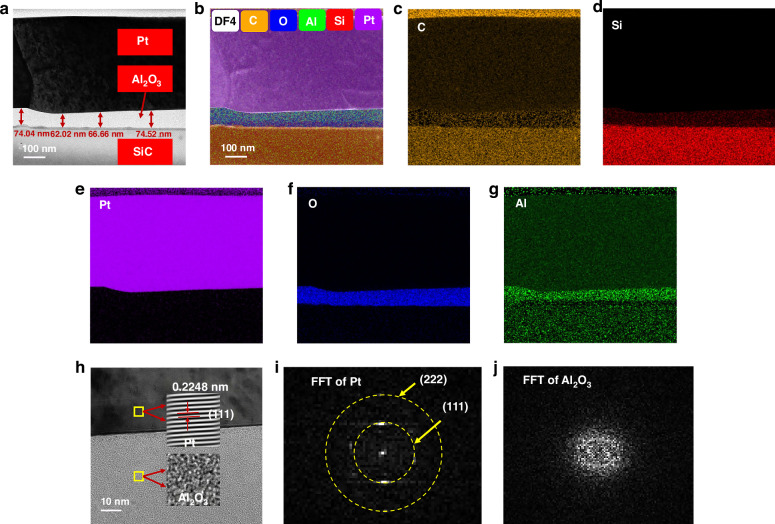
Images and elemental analyses of the cross-sections of Al_2_O_3_/Pt multilayers observed in HAADF mode. **a** Cross-sectional image of a multilayer film. **b** Various element distributions across the entire cross-section. **c**–**g** Element distribution across the entire cross-section. **h** Crystal structures of the Al_2_O_3_ film and the Pt film. **i**, **j** FFT analyses of the high-resolution images of a Pt thin film and an Al_2_O_3_ thin film on a cross-section

To elucidate the interfacial diffusion between the Pt film and the substrate, the SiC/Al_2_O_3_/Pt multilayer slice (after annealing at 950 °C for 1.5 h in air) was observed via Lorentz transmission electron microscopy. As shown in the high-resolution image in Fig. 2a, the cross-section of the Al_2_O_3_/Pt multilayer film was clear and smooth. Figure 2b shows the distributions of the various elements on the cross-section, and Fig. 2c–g illustrates the distributions of the elements in the cross-section. As observed from the distributions of Al, O and Pt, no diffusion occurred between the Al_2_O_3_ layer and the Pt layer. The interface between the Al_2_O_3_ film and Pt film was stable. Figure 2h shows the crystal structures of the alumina film and the Pt film. Figure 2i, j shows the fast Fourier transform (FFT) analyses of high-resolution images of the cross-sections of the Pt thin film and Al_2_O_3_ thin film, respectively. The Pt film had (111) and (222) crystal planes, and its (111) crystal plane spacing was 0.2248 nm. However, the Al_2_O_3_ film was polymorphic. As demonstrated by the experimental results in this section, no mutual diffusion occurred between the Pt film and the SiC substrate after aging in air at 950 °C for 1.5 h by adding the Al_2_O_3_ layer.

The thicknesses of the Al_2_O_3_ layers were measured at four arbitrary positions, as annotated in Fig. 2a. From left to right, the thicknesses of the Al_2_O_3_ were 74.04, 62.02, 66.66, and 74.52 nm. The average thickness was 69.31 nm, with a standard deviation of 5.24 nm. This variation in thickness was attributed to process errors during sputtering of the Al_2_O_3_ film, leading to nonuniformity; however, the variation was acceptable. If the Al_2_O_3_ layer disappeared, an interfacial reaction occurred between SiC and Pt above 500 °C, resulting in the formation of Pt and Si compounds, which degraded the electrical performance of Pt, as investigated in the literature^30–34^.

#### Microtopography analysis

**Fig. 3:**
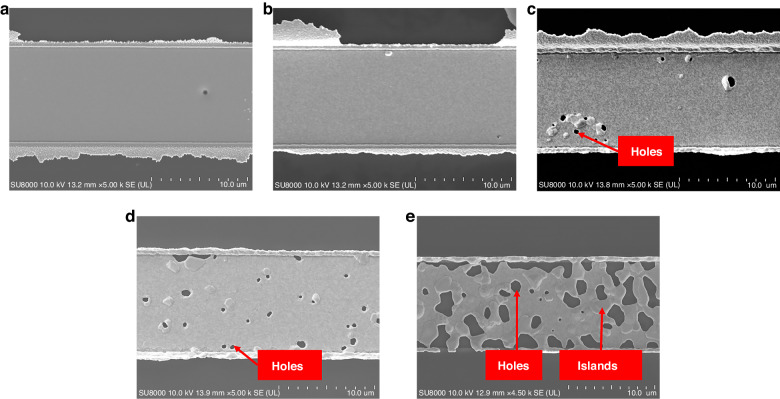
SEM surface micrographs of Al_2_O_3_/Pt films annealed in air at different temperatures. **a** As-deposited. **b** Annealed at 650 °C. **c** Annealed at 750 °C. **d** Annealed at 850 °C. **e** Annealed at 950 °C

**Fig. 4:**
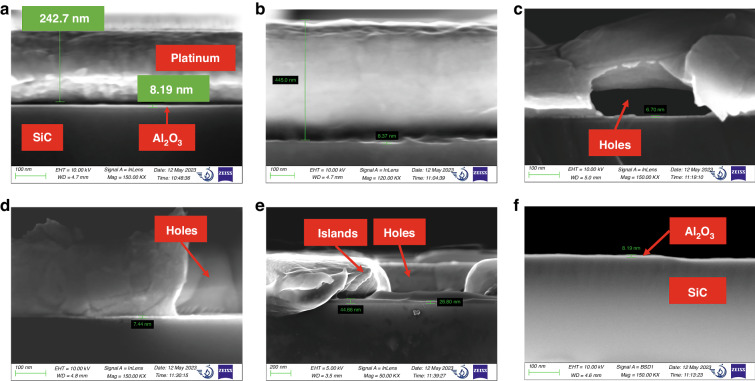
SEM images of the cross-sections of SiC/Al_2_O_3_/Pt multilayers annealed at different temperatures. **a** As-deposited. **b** Annealed at 650 °C. **c** Annealed at 750 °C. **d** Annealed at 850 °C. **e** Annealed at 950 °C. **f** Cross-sectional image of SiC/Al_2_O_3_ in backscatter mode

Surface topography characterization and cross-sectional morphology characterization by SEM were performed on Al_2_O_3_/Pt films to explore the effect of annealing temperature on the quality of the Pt film. Figure 4a shows that the thickness of the Pt film was 242.7 nm. Figure 4f, which was captured in the backscatter mode of FE-SEM, illustrates the presence of a thin layer of Al_2_O_3_ on the SiC substrate. This layer had a thickness of 8.19 nm. As shown in Fig. 3a and Fig. 4a, the surface of the deposited Pt film appeared dense, and the cross-section was dense and uniform, indicating the good quality of the deposited film. After annealing at 650 °C, the surface of the Pt film remained dense, as shown in Fig. 3b. Moreover, the cross-section of the Pt film remained dense and uniform, as shown in Fig. 4b. After annealing at 750 °C, holes began to appear but did not extensively erode the surface of the film, as illustrated in Figs. 4c and 3c. As the annealing temperature increased to 850 °C, the holes completely pierced the Pt film in the thickness direction, as depicted in Fig. 4d. Additionally, the number of holes increased, and microscale ridges and valleys emerged on the surface of the Pt film, as shown in Fig. 3d. As the annealing temperature increased to 950 °C, the holes were enlarged, causing the film to break into islands, as shown in Fig. 3e. This observation aligned with the phenomenon depicted in Fig. 4e, indicating that the holes grew and merged, creating “islands”. Similar results were observed for both Ti/Pt and Ta/Pt films by SL Firebaugh et al.^18^. It was determined that by increasing the annealing temperature above 750 °C, the surface quality of the Pt film deteriorated. The appearance of holes and islands was caused by the self-diffusion of Pt and thermal stress^18^.

**Fig. 5:**
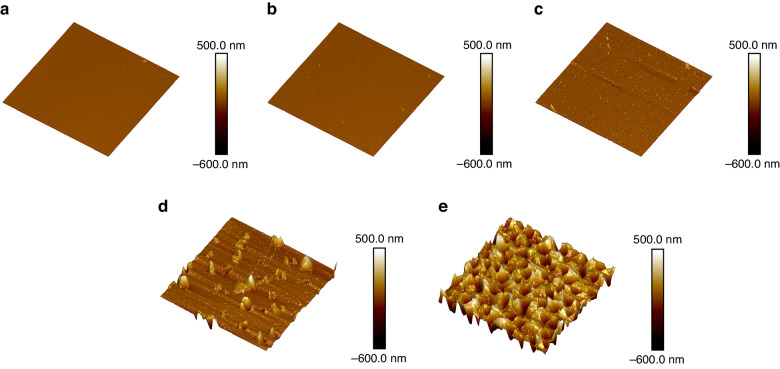
3D AFM surface topographies of Al_2_O_3_/Pt films deposited on SiC substrates annealed at different temperatures. **a** As-deposited. **b** Annealed at 650 °C. **c** Annealed at 750 °C. **d** Annealed at 850 °C. **e** Annealed at 950 °C

To evaluate the deterioration of the surface quality of the Pt film and to elucidate the deterioration mechanism, the particle size and pore density with increasing annealing temperature of the film were characterized using AFM. The scanning results are shown in Fig. 5. Table 1 shows the results of the scanning images processed by the NanoScope Analysis software. *R*_a_ and *R*_q_ represent the absolute mean deviation and the root mean square deviation of the surface roughness, respectively, as listed in Table 1. The smallest *R*_a_ and *R*_q_ values were observed for the as-deposited films. The film roughness first increased when annealed at 650 °C because of Pt recrystallization and then increased sharply from 750 to 950 °C because of the agglomeration and coalescence of the grains. In addition, the average particle diameter and pore density are provided in Table 1. There were no obvious out-of-plane convex particles on the surfaces of the as-deposited Pt film and the samples annealed at 650 °C. These results were consistent with those shown in Fig. 5a, b. When the annealing temperature reached 750 °C, out-of-plane convex particles and pores appeared on the surface of the film, with an average particle size of 0.111 µm and a pore density of 0.844%. The formation of these bulges and pores were similar to that of holes reported in published articles^35–37^. Holes appeared because the force driving grain growth caused by the annealing temperature of the Pt film was overly high, resulting in film coalescence and agglomeration. Therefore, when the annealing temperature reached 850 °C, the grains of the Pt film quickly coalesced, and some of them formed islands. Furthermore, film agglomeration was obvious, and the pore density increased to 1.14%. When the annealing temperature reached 950 °C, the agglomeration of the film was severe, and the pore density reached 19.88%. Thus, the surface of the Pt film became rough as the annealing temperature increased, resulting in a decrease in the TCR of the Pt thin-film RTD and a decrease in the average linearity of the three R–T curves, as shown in Table S4.

**Table 1:** Surface morphologies of the Al_2_O_3_/Pt samples annealed at different temperatures by AFM

Annealing temp. (°C)	*R*_a_ (nm)	*R*_q_ (nm)	Grain diameter (µm) (particle analysis)	Hole density (bearing analysis)
As-deposited	1.43	2.21	None	None
650	1.59	2.68	None	None
750	4.25	8.78	0.111	0.844%
850	27.2	50.6	1.266	1.14%
950	127	160	1.723	19.88%

Note: None indicates that no significant particles or holes appeared

#### Microstructural analysis

**Fig. 6:**
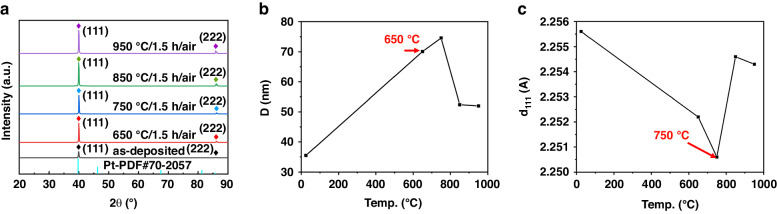
XRD Characterization of the Pt films annealed at different temperatures. **a** XRD patterns of Pt films annealed at different temperatures. **b** Annealing temperature-dependent grain size D_111_ obtained by XRD. **c** Pt (111) lattice spacing d_111_ determined by XRD

The grain size and crystal plane spacing were obtained from the XRD pattern to microscopically reveal the deterioration mechanism of the surface quality of the Pt film. Figure 6a shows the XRD pattern of the Pt film. The (111) crystal plane was the preferential orientation, and the (111) peak strength increased with increasing annealing temperature. Figure 6b shows that the grain size of the Pt thin film depended on the annealing temperature. The grain size listed in Table 2 was calculated by considering the broadening of the (111) crystal plane according to Eq. 2. For the as-deposited Pt film, the grain size was 35.5 nm. The grain size increased with increasing annealing temperature, from 70.1 nm at 650 °C to 74.6 nm at 750 °C. At 750 °C, the grain size peaked because of secondary recrystallization. After annealing at temperatures greater than 750 °C, the grain size decreased to 52.4 nm at 850 °C and then reached a minimum of 52 nm at 950 °C. The crystal plane spacing d_111_^23^ was used to characterize the residual stress in the plane of the Pt film. The in-plane compressive stress led to perpendicular lattice expansion, whereas the in-plane tensile stress led to perpendicular lattice contraction. The changes in d_111_ with annealing temperature are shown in Fig. 6c. For bulk Pt, the standard d_111_ was 2.265 Å, while the d_111_ of the as-deposited Pt film in this study was 2.2556 Å, implying that the as-deposited Pt film experienced tensile stress. This phenomenon differed from the findings in the literature, where the as-deposited Pt films exhibited compressive stress^25^. The reason for this stress was that the working pressure was greater in this study. The decrease in d_111_ indicated an increase in stress with increasing annealing temperature. At 750 °C, d_111_ reached a minimum value of 2.2506 Å, and the residual stress of the Pt film peaked. At this temperature, the residual tensile stress of the Pt film reached the strength limit and was subsequently relieved by plastic deformation. Accordingly, Pt atoms moved along the grain from the interior to the film surface boundary, resulting in the formation of hillocks. Additionally, the Tammann temperature (*T*_m_) of Pt (defined as 0.5 *T*_*M*_, where *T*_*M*_ is the melting point of the material, expressed in Kelvin) was approximately 750 °C, at which point self-diffusion occurred for metallic materials. At the microscale, under these conditions, the atomic mobility of the material was high^38^. As the temperature exceeded 750 °C, the migration of Pt atoms accelerated, resulting in additional pores in the Pt films and the formation of islands. This phenomenon was consistent with the surface topography characterization results of the Pt film. The self-diffusion rate of Pt films was closely related to the temperature, oxygen, and adhesion of the Pt film to the substrate, all of which greatly affected the agglomeration of Pt^18^. It was inferred that the tensile stress and self-diffusion of Pt films resulted in the formation of hillocks.

**Table 2:** Grain size and crystal plane spacing of the Pt films under different conditions

Annealing temp. (°C)	Grain size D (nm)	Crystal plane spacing d_111_ (Å)
As-deposited	35.5	2.2556
650/1.5 h/air	70.1	2.2522
750/1.5 h/air	74.6	2.2506
850/1.5 h/air	52.4	2.2546
950/1.5 h/air	52	2.2543

### Electrical properties of the Pt thin-film RTD

The electrical properties of the Pt thin-film RTD were tested in this section to evaluate the effect of annealing on the sensing performance of the Pt thin-film RTD. The optimal annealing process conditions were obtained.

#### Effect of thermal annealing on the resistivity of the Pt thin-film RTD

**Fig. 7:**
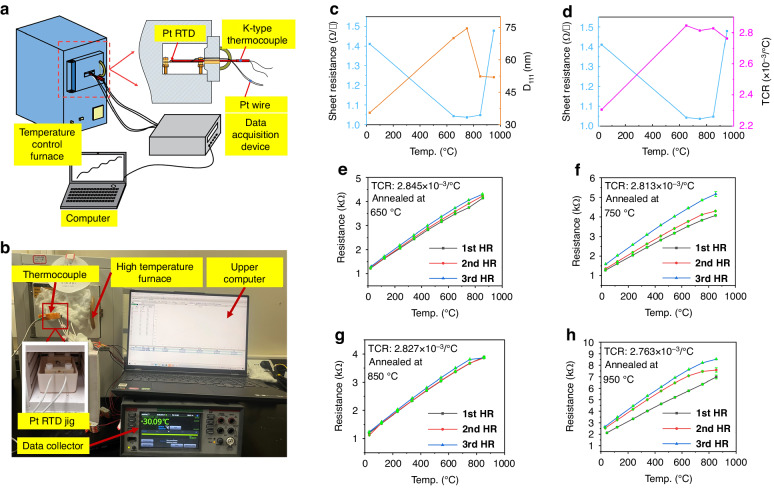
Performance tests of the Pt thin films. **a** Schematic diagram of the temperature–resistance test platform. **b** Temperature–resistance test platform built in the laboratory. **c** Sheet resistance and grain size D_111_ values of Pt thin films under different annealing temperatures. **d** Sheet resistance and TCR values of Pt thin films under different annealing temperatures. **e**–**h** Temperature–resistance curves and calculated TCRs of Pt thin-films annealed for 1.5 h at different annealing temperatures in air

The variations in the square resistance and grain size of the Pt film as functions of annealing temperature are shown in Fig. 7c. The obtained square resistance at each annealing temperature was the average of three independent measurements. Notably, Fig. 7c reveals that annealing temperatures below 750 °C effectively reduced the square resistance of the Pt film. This reduction was accompanied by an increase in the grain size D_111_. This change suggested a strong correlation between the reduction in the resistivity of the Pt thin film and the growth of its grains. Grain growth is typically associated with the annihilation of voids, a reduction in dislocation density^39^, and a decrease in grain boundaries^40^. According to Matthiessen’s rule^41^, reductions in defects and grain boundaries decreased the resistivity of Pt films. When the annealing temperature exceeded 750 °C, the square resistance of the Pt film began to increase from 1.037 Ω per square at 750 °C to 1.048 Ω per square at 850 °C and reached a maximum of 1.479 Ω per square at 950 °C. This increase in the square resistance was attributed to the agglomeration of Pt film, which deteriorated the surface quality of the film.

#### Effect of thermal annealing on the TCR of the Pt thin-film RTD

The effect of annealing temperature on the TCR of the Pt thin-film RTD was investigated in this section. The samples were annealed in air for 1.5 h at 650 °C, 750 °C, 850 °C and 950 °C. Figure 7a shows a schematic of a high-temperature test platform for Pt thin-film RTD. Figure 7b shows a high-temperature experimental system built in the laboratory for Pt thin-film RTD. The temperature of the furnace was calibrated in the temperature range of 200–900 °C by an external K-type armored thermocouple with an accuracy of 0.034 °C. Figure 7e–h shows the temperature–resistance curves of the Pt thin-film RTD at temperatures ranging from 25 to 850 °C. A total of 12 sensors was used. Every 3 samples with very close resistance values were selected for each annealing temperature (650, 750, 850, and 950 °C) and then tested. Each sensor underwent three heating ramp (HR) tests. Since the differences between the three samples at each annealing temperature were small, the test data of only one sample were provided here. The average TCR, maximum operating temperature, and goodness-of-fit were calculated from the three temperature increase tests, as shown in Table 3. The changes in the TCR of the Pt thin-film RTD with annealing temperature are shown in Fig. 7d. Below 650 °C, the resistivity of the Pt film was inversely proportional to the TCR, as reported in other literature^42^. Therefore, to obtain an increased TCR, it was necessary to reduce the resistivity of the Pt film. However, due to the appearance of holes and islands in the Pt film at temperatures of at least 750 °C, the resistivity of the Pt film was not related to the TCR. As shown in Table 3 and Fig. 7e–h, annealing generally improved the TCR and linearity of the Pt thin-film RTD. Specifically, with increasing annealing temperature, the TCR reached a maximum value of 2.845 × 10^−3^/°C at 650 °C. This peak occurred due to the growth of grains and the decrease in the number of grain defects in the Pt film at temperatures below 650 °C. However, the TCR decreased when annealing at 750 °C due to the appearance of pores in the Pt film. During annealing at 850 °C, the TCR was greater than that at 750 °C. One possible reason for this phenomenon was the increased thicknesses of the Pt film islands caused by agglomeration^42^. For annealing at 750 °C and 950 °C, the repeatability and linearity of the resistance–temperature curves were poor. Considering the data in Table 3 and the repeatability of the Pt thin-film RTD data in Fig. 7, we believed that the optimal annealing temperature for the Pt films was 650 °C, at which point the TCR and linearity of the Pt thin-film RTD were both high.

**Table 3:** Electrical properties of Pt films subjected to RTD after annealing in air for 1.5 h at different annealing temperatures

Annealing temp. (°C)	Square resistance (Ω/□)	TCR (×10^−3^/°C)	Maximum temp. (°C)	*R*^2^
As-deposited	1.411	2.305	856.595	0.83766
650/1.5 h/air	1.043	2.845	855.108	0.99719
750/1.5 h/air	1.037	2.813	852.608	0.99569
850/1.5 h/air	1.048	2.827	853.627	0.99155
950/1.5 h/air	1.479	2.763	853.791	0.98818

**Fig. 8:**
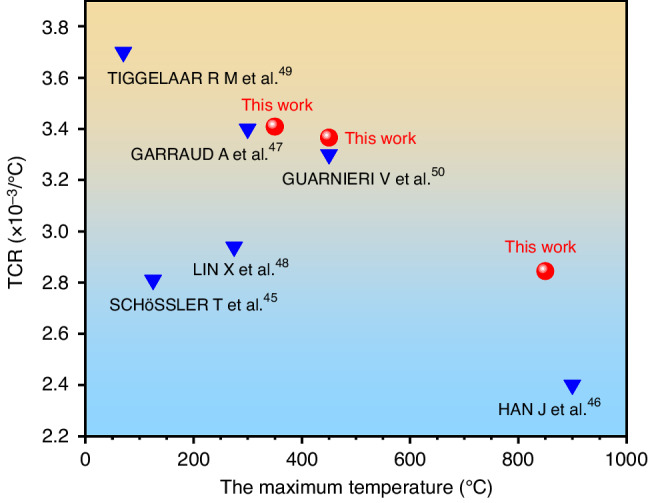
Comparision of maximum working temperature and TCR between this work and previous studies. Maximum working temperature and TCR values of Pt films subjected to RTD in this study compared to those reported in the literature^23,44–48^

The Al_2_O_3_/Pt bilayer film with a Pt thickness of 242.7 nm that was prepared on a SiC substrate had excellent electrical properties after annealing in air at 650 °C. When the thickness of the Pt film increased, the TCR further increased. Compared with the results reported in previous literature, the Pt thin-film RTD prepared in this study had a wide working range, reaching 850 °C. Additionally, the TCR of the Pt thin-film RTD prepared in this study was higher than that obtained in the same working range, as shown in Fig. 8. The major reason for the difference in this work from the literature was that there was no mutual diffusion between Pt and the substrate.

As seen in the T–R test results in Fig. 7e–g, the Pt thin-film temperature sensor based on the SiC substrate underwent three consecutive heating tests, with temperatures reaching 850 °C. The sensor exhibited a high temperature coefficient of resistance (TCR) and good linearity, indicating its excellent performance in high-temperature environments, which laid the foundation for on-chip integration with SiC pressure sensors for temperature compensation. Based on the high-temperature pressure testing curves of the SiC pressure sensor previously published by our group^43^, it was evident that the zero-pressure output and full-scale output varied significantly with temperature, indicating significant temperature drift in the pressure sensor. To improve the accuracy of the pressure sensor, it was necessary to monitor the on-chip temperature and perform real-time compensation to eliminate the influence of temperature on the pressure sensor output.

#### Relationships among microscopic quality, surface morphology, and electrical properties

**Fig. 9:**
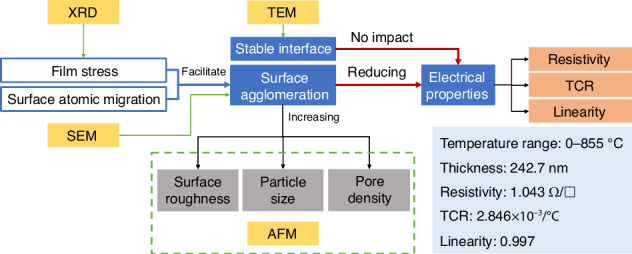
Summary of the effects of microscale parameters on properties of Pt thin films. Schematic diagram of the influences of microscopic parameters on the surface morphological and electrical properties of Pt films

The relationships among the microscopic stress, surface morphological, and electrical properties of the Pt films are presented in Fig. 9. The optimized performance of the Pt RTD in this study was provided. Notably, the Al_2_O_3_/Pt bilayer film prepared in this study maintained interfacial stability at 950 °C in air. Thus, the high-temperature electrical properties of the Pt RTD films were not affected by interfacial diffusion but were only related to surface agglomeration. The surface agglomeration of Pt films appeared in the form of sharp increases in surface roughness, particle size and pore density, which reduced the electrical properties, including resistivity and TCR, and the output linearity of Pt thin-film RTDs. The thermal stress and self-diffusion levels of Pt films were the forces driving film agglomeration. Therefore, reducing the self-diffusion coefficient and reasonably controlling the stress of the film was highly important for reducing agglomeration and improving the high-temperature electrical properties of Pt films RTD.

The Pt thin-film temperature detector based on a SiC substrate developed in this paper laid the foundation for future on-chip integration with SiC pressure sensors, thereby enabling temperature compensation for the pressure sensor. Based on the high-temperature pressure testing results of the SiC pressure sensor in our previously published work^43^, it was evident that the zero-pressure output and full-scale output varied significantly with temperature, indicating significant temperature drift in the pressure sensor output at high temperatures. To improve the accuracy of the pressure sensor, it was necessary to monitor the on-chip temperature and perform real-time compensation to eliminate the influence of temperature on the pressure sensor output. The Pt thin-film temperature detector in this study was designed to perform temperature monitoring, i.e., in situ temperature monitoring of SiC pressure sensor chips, in the future. When SiC pressure sensors operated in unknown high-temperature environments, temperature fluctuations caused significant drifts in the pressure sensor outputs, reducing the pressure measurement stability and accuracy. Therefore, for temperature compensation, it was necessary to accurately monitor the temperatures of SiC pressure sensor chips and to calibrate and eliminate the effect of temperature on the pressure sensor output to obtain an accurate output value corresponding to the loaded pressure.

## Conclusion

In this paper, we proposed a novel microfabrication method for fabricating Pt thin-film RTDs anchored in AlN grooves with additional Al_2_O_3_ layers. This fabrication technique successfully produced Pt-film RTDs on SiC substrates. At high temperatures, specifically 950 °C, the Pt thin-film RTD demonstrated strong adhesion to the substrate. The Al_2_O_3_/Pt bilayer film developed in this study exhibited a stable interface at 950 °C, which provided a solid basis for the high-temperature electrical performance of the Pt thin-film RTD. The maximum operating temperature exceeded 850 °C, with a TCR of 2.845 × 10^−3^/°C and high linearity, as demonstrated by the experimental results.

This research established a foundational approach for integrating Pt-film RTDs with SiC pressure sensors on a single chip. Furthermore, in-depth analyses of the changes in interfacial diffusion, microstructure, microscopic stress, and electrical properties of Pt films at various annealing temperatures were provided. This study established relationships between these factors and the electrical performance characteristics of Pt thin-film RTDs. Notably, the Al_2_O_3_/Pt bilayer film maintained a stable interface after annealing at 950 °C. There was no evidence of elemental diffusion. Consequently, any deterioration in the surface quality of the Pt film was not attributed to the Al_2_O_3_ layer.

The study revealed that below 650 °C, grain recrystallization, and growth reduced the numbers of defects and grain boundaries in the Pt film, thereby decreasing the resistivity and increasing the TCR. The initial stress in the as-deposited Pt film acted as tensile stress, which increased with increasing annealing temperature. At 750 °C, the stress in the Pt film reached its strength limit. At approximately this temperature, close to the Tammann temperature of the Pt film, the Pt atoms exhibited high mobility, leading to the formation of holes in the film. This phenomenon intensified with increasing annealing temperature, eventually resulting in the formation of isolated islands at 950 °C, thereby disrupting the continuity of the film. This deterioration in the surface quality of the Pt film substantially reduced the TCR and linearity of the Pt thin-film RTD. Therefore, the annealing temperature of the film should preferably be below 750 °C. The optimum annealing temperature in this paper was 650 °C. To further enhance the temperature resistance of the Pt film, appropriate measures should be taken to suppress the agglomeration of the Pt film.

## Supplementary information

Supplemental material
