# Supplementary material for: Pt thin-film resistance thermo detectors with stable interfaces for potential integration in SiC high-temperature pressure sensors
Source: Microsyst Nanoeng. 2024 Sep 26;10:133. doi: 10.1038/s41378-024-00746-w (PMC11427678; doi:10.1038/s41378-024-00746-w)
Supplement: Supplementary file 1 — Supplemental material [file 41378_2024_746_MOESM1_ESM.docx]

***Supporting Information***

**Pt Thin Film Resistance Thermo Detectors with Stable Interface for potential integration in SiC High Temperature Pressure Sensors**

Ziyan Fang^1,2,3,4^, Xiaoyu Wu^2^*, Hu Zhao^2*^, Xudong Fang^1,2,3,4*^, Chen Wu^1,2^, Dong Zhang^1,2^, Zhongkai Zhang^1,2,3,4^, Bian Tian^1,2,3,4^,Libo Zhao^1,,3,4,5^, Tiefu Li^6^, Prateek Verma^7^, Ryutaro Maeda^1,5^ and Zhuangde Jiang^1,2^

*^1^State Key Laboratory for Manufacturing Systems Engineering, International Joint Laboratory for Micro/Nano Manufacturing and Measurement Technologies, Xi’an Jiaotong University, Xi’an 710049, China*

*^2^School of Mechanical Engineering, Xi’an Jiaotong University, Xi’an 710049, China*

*^3^Shandong Laboratory of Yantai Advanced Materials and Green Manufacturing* *at Yantai, Yantai 264000, China*

*^4^Xi’an Jiaotong University (Yantai) Research Institute for Intelligent Sensing Technology and System*

*^5^School of Instrument Science and Technology, Xi’an Jiaotong University, Xi’an 710049, China*

*^6^School of Integrated Circuits, Tsinghua University, Beijing 100084, China,*

*^7^Department of Chemical Engineering, University of Arkansas, Fayetteville, AR 72701, USA*

**E-mail:* Xiaoyu Wu ([XYmems@stu.xjtu.edu.cn](mailto:XYmems@stu.xjtu.edu.cn)), Hu Zhao ([zhaoh087@avic.com](mailto:zhaoh087@avic.com)), Xudong Fang ([dongfangshuo30@xjtu.edu.cn](mailto:dongfangshuo30@xjtu.edu.cn) )

Table S1. The thickness of the aluminum oxide layer at four arbitrary positions in Fig. 2a, along with the average thickness and standard deviation.

| Thickness  1(nm) | Thickness  2(nm) | Thickness  3(nm) | Thickness  4(nm) | Average value(nm) | Standard deviation |
| --- | --- | --- | --- | --- | --- |
| 74.04 | 62.02 | 66.66 | 74.52 | 69.31 | 5.24 |

In Fig. 2a in the manuscript, we measured the thickness of the aluminum oxide layer at four arbitrary positions and annotated in the Fig. 2a. From left to right, the thickness of aluminum oxide was as follows: 74.04 nm, 62.02 nm, 66.66 nm, and 74.52 nm. The average thickness was 69.31 nm, with a standard deviation of 5.24 nm. The corresponding values were listed in Table S1. This variation in thickness was attributed to process errors during sputtering the aluminum oxide film, leading to non-uniformity, however, the variation is acceptable.


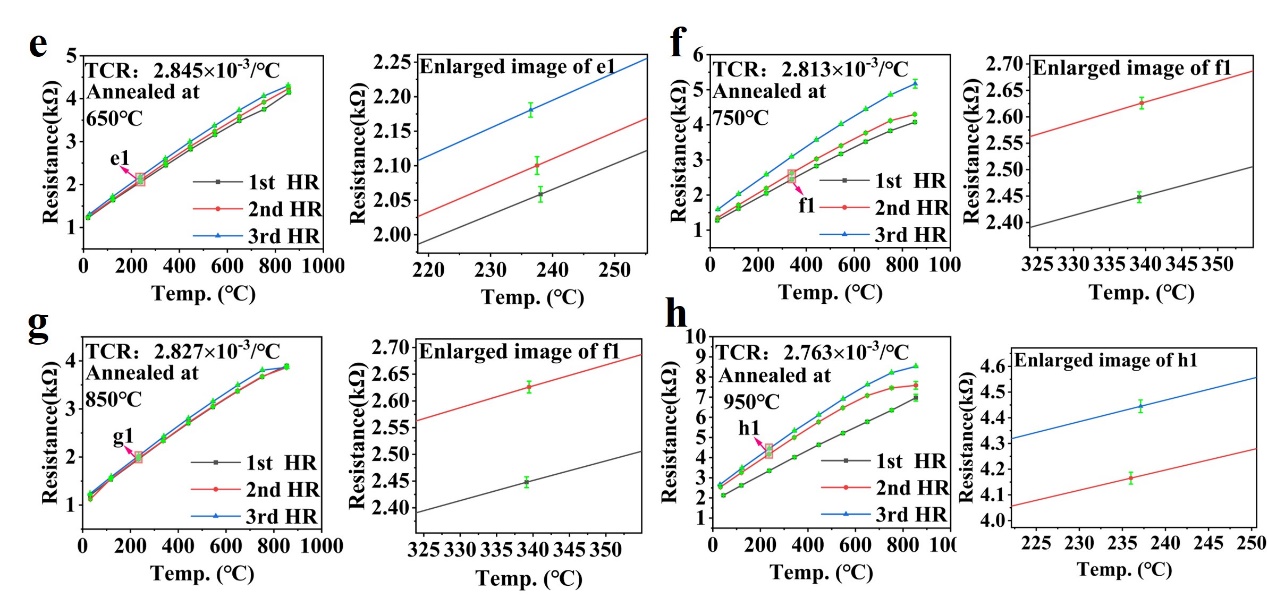


Fig. S1 e, f, g, h The partial enlarged image of the error bars for the sample annealed for 1.5 h at different annealing temperatures.

Fig. S1 e, f, g, h showed the partial enlarged image of the error bars, corresponding to the Fig. 7e, f, g, h in the manuscript. And also, the standard deviations of the resistance values at each temperature point at different annealing temperatures were listed in Table S2.

Table S2. The standard deviation of the resistance (R) corresponding to each temperature point in the Fig. 7e, f, g, h.

| Annealed at 650℃ for 1.5 hour in air | | | | | |
| --- | --- | --- | --- | --- | --- |
| 1st HR | | 2nd HR | | 3rd HR | |
| Resistance  (R)（Ω） | Standard deviation of R | Resistance  (R) | Standard deviation of R | Resistance  (R) | Standard deviation of R |
| 1224.778 | 0.101 | 1244.45 | 0.155 | 1309.41 | 0.319 |
| 1639.113 | 6.176 | 1649.69 | 7.975 | 1717.751 | 6.977 |
| 2058.572 | 11.228 | 2100.251 | 12.849 | 2180.808 | 10.407 |
| 2447.253 | 5.511 | 2503.568 | 7.419 | 2599.847 | 7.833 |
| 2821.66 | 3.069 | 2884.38 | 5.241 | 2993.654 | 3.725 |
| 3159.794 | 7.921 | 3241.934 | 6.437 | 3368.026 | 5.062 |
| 3484.727 | 3.501 | 3586.989 | 4.74 | 3729.681 | 3.493 |
| 3752.136 | 3.162 | 3917.37 | 5.391 | 4059.465 | 2.815 |
| 4146.456 | 8.024 | 4233.731 | 33.046 | 4304.064 | 18.439 |

| Annealed at 750℃ | | | | | |
| --- | --- | --- | --- | --- | --- |
| 1st HR | | 2nd HR | | 3rd HR | |
| Resistance  (R)（Ω） | Standard deviation of R | Resistance  (R) | Standard deviation of R | Resistance  (R) | Standard deviation of R |
| 1278.829 | 0.158 | 1359.49 | 0.154 | 1359.49 | 0.154 |
| 1614.878 | 8.955 | 1719.584 | 9.926 | 1719.584 | 9.926 |
| 2048.051 | 13.364 | 2191.875 | 15.895 | 2191.875 | 15.895 |
| 2447.914 | 10.124 | 2625.983 | 11.014 | 2625.983 | 11.014 |
| 2824.851 | 5.877 | 3030.729 | 6.294 | 3030.729 | 6.294 |
| 3173.097 | 12.793 | 3406.62 | 9.972 | 3406.62 | 9.972 |
| 3522.183 | 7.171 | 3768.964 | 9.15 | 3768.964 | 9.15 |
| 3830.744 | 4.298 | 4118.109 | 17.64 | 4118.109 | 17.64 |
| 4072.395 | 3.624 | 4288.623 | 14.148 | 4288.623 | 14.148 |

| Annealed at 850℃ | | | | | |
| --- | --- | --- | --- | --- | --- |
| 1st HR | | 2nd HR | | 3rd HR | |
| Resistance  (R)（Ω） | Standard deviation of R | Resistance  (R) | Standard deviation of R | Resistance  (R) | Standard deviation of R |
| 1202.429 | 1.346 | 1121.951 | 0.106 | 1235.058 | 0.26 |
| 1533.046 | 7.86 | 1543.081 | 7.62 | 1588.138 | 6.502 |
| 1954.134 | 11.107 | 1970.919 | 11.176 | 2023.558 | 12.02 |
| 2339.68 | 6..149 | 2336.009 | 12.962 | 2426.128 | 6.147 |
| 2701.571 | 3.047 | 2720.013 | 1.334 | 2802.573 | 3.276 |
| 3041.105 | 4.355 | 3056.539 | 4.477 | 3155.347 | 4.618 |
| 3366.201 | 3.175 | 3378.731 | 2.156 | 3494.126 | 2.716 |
| 3665.338 | 1.465 | 3676.66 | 4.583 | 3807.139 | 4.86 |
| 3892.073 | 8.957 | 3868.018 | 8.551 | 3827.803 | 16.147 |

| Annealed at 950℃ | | | | | |
| --- | --- | --- | --- | --- | --- |
| 1st HR | | 2nd HR | | 3rd HR | |
| Resistance  (R)（Ω） | Standard deviation of R | Resistance  (R) | Standard deviation of R | Resistance  (R) | Standard deviation of R |
| 2128.577 | 4.138 | 2538.698 | 0.576 | 2661.895 | 0.235 |
| 2619.458 | 11.924 | 3256.706 | 16.316 | 3479.942 | 14.905 |
| 3349.103 | 16.533 | 4165.258 | 23.171 | 4444.785 | 24.657 |
| 4016.797 | 10.093 | 4999.08 | 14.088 | 5326.847 | 15.783 |
| 4636.022 | 4.342 | 5767.438 | 7.212 | 6114.399 | 7.797 |
| 5212.479 | 11.572 | 6468.41 | 14.441 | 6908.895 | 10.715 |
| 5770.056 | 9.082 | 7076.932 | 8.54 | 7620.167 | 7.433 |
| 6353.423 | 27.649 | 7457.029 | 57.522 | 8209.401 | 15.149 |
| 6972.258 | 167.212 | 7586.34 | 188.021 | 8527.84 | 18.437 |

Table S3. The sensors used in this manuscript and its annealing conditions.

|  | Sensor #1 | Sensor #2 | Sensor #3 | Sensor #4 |
| --- | --- | --- | --- | --- |
| Annealing conditions | 650℃ for 1.5 hour in air | 750℃ for 1.5 hour in air | 850℃ for 1.5 hour in air | 950℃ for 1.5 hour in air |
| Initial resistance value(Ω) | 1224.778 | 1278.829 | 1202.429 | 2128.577 |

Table S3 showed the sensors used in this manuscript, a total of 12 sensors were used. Every 3 samples with very close resistance values were selected for each annealing temperature (i.e. 650 °C, 75 0 °C, 850 °C, and 950 °C respectively) and were then tested. Each sensor underwent three heating ramp (HR) tests. Since the difference between the 3 samples at each annealing temperature was small, the test data of only one sample was provided here. The design resistance of all four sensors was 1000 ohms. The difference in initial resistance values in the table was reasonable caused by different annealing conditions.

Table S4. The electrical properties varied with the roughness of Ra.

| Ra(nm) | TCR(×10^-3^/℃) | R^2^ |
| --- | --- | --- |
| 1.59 | 2.845 | 0.99719 |
| 4.25 | 2.813 | 0.99569 |
| 27.2 | 2.827 | 0.99155 |
| 127 | 2.763 | 0.98818 |

Table S4 showed the degradation in the electrical performance of the Pt thin film, namely a decrease in TCR, and a decrease in averaged linearity of the three tests R-T curve with the increasing of the thin film surface roughness.
